# Humoral response to mRNA vaccines against SARS-CoV-2 in patients with humoral immunodeficiency disease

**DOI:** 10.1371/journal.pone.0268780

**Published:** 2022-06-09

**Authors:** Michaela Bitzenhofer, Franziska Suter-Riniker, Matthias B. Moor, Daniel Sidler, Michael P. Horn, Anna Gschwend, Cornelia Staehelin, Andri Rauch, Arthur Helbling, Lukas Jörg

**Affiliations:** 1 Division of Allergology and clinical Immunology, Department of Pneumology and Allergology, Inselspital, Bern University Hospital, University of Bern, Bern, Switzerland; 2 Clinical Microbiology, Institute for Infectious Disease, University of Bern, Bern, Switzerland; 3 Department of Nephrology and Hypertension, Inselspital, Bern University Hospital, University of Bern, Bern, Switzerland; 4 Department of Clinical Chemistry, Inselspital, Bern University Hospital, University of Bern, Bern, Switzerland; 5 Department of Infectious Diseases, Inselspital, Bern University Hospital, University of Bern, Bern, Switzerland; 6 Department of Dermatology, Allergy Unit, University Hospital of Zurich, Zurich, Switzerland; University of Hail, SAUDI ARABIA

## Abstract

**Objectives:**

Although mRNA-based vaccines against SARS-CoV-2 induce a robust immune response and prevent infections and hospitalizations, there are limited data on the antibody response in individuals with humoral immunodeficiency. The aim of this study was to evaluate the humoral immune response after two vaccine doses with BNT162b2 or mRNA-1273 in patients with humoral immunodeficiency disease.

**Methods:**

This cross-sectional study assessed 39 individuals with hypogammaglobulinemia under immunoglobulin replacement therapy. IgG anti-SARS-CoV-2 spike protein antibodies (anti-S) were measured 4 weeks to 4 months after two doses of an mRNA vaccine against SARS-CoV-2. The proportion of patients, who developed a humoral immune response to the spike protein were evaluated and compared to 19 healthy controls.

**Results:**

After vaccination with two vaccine doses, 26/39 patients (66.7%) with humoral immunodeficiency disease and all healthy controls developed anti-S. In subjects with baseline IgG <3 g/l, only 1/5 (20%) showed a humoral immune response. 10 out of 26 with CVID (38.5%) and 7/9 under immunosuppressive drugs (77.8%) developed no immune response (13 subjects with no response) compared to 0/19 in healthy controls. Subgroup analysis in patients without immunosuppressive drugs revealed lower anti-S in patients with moderate to severe humoral immunodeficiency disease: baseline IgG <3 g/l: 12.0 AU/ml (95%CI 12.0–125.0), baseline IgG 3–5 g/l: 99.9 AU/ml (95%CI 14.4–400.0), baseline IgG >5 g/l: 151.5 AU/ml (95%CI 109.0–400.0), healthy controls 250.0 AU/ml (95%CI 209.0–358.0), p = 0.007.

**Conclusion:**

In most patients with mild to moderate humoral immunodeficiency we found only slightly lower anti-S antibodies compared with healthy controls after two vaccine doses with BNT162b2 and mRNA-1273. However, in patients with a decreased baseline IgG below 3 g/l and/or under immunosuppressive drugs, we found severely impaired humoral immune responses.

## Introduction

Following the global spread of severe acute respiratory syndrome coronavirus 2 (SARS-CoV-2), vaccinations have been introduced since the end of 2020 to control the pandemic. In particular, mRNA-based vaccines are considered highly effective in prevention of infection and hospitalization, even against variants of concern [1,2]. To date, data on the humoral immune response to SARS-CoV-2 in patients with immunodeficiency disorders is limited [3]. Due to hypogammaglobulinemia and T- and B-cell impairment, the general immune response is reduced in these patients, which may explain severe or fatal Covid-19 infections in this population [3–5]. In Switzerland, this population was prioritized for vaccination early in 2021 with mRNA vaccines. In recent months, an increasing number of patient groups have been identified which do not have an optimal vaccine response to the Covid-19 vaccines. After two doses of Covid-19 vaccine, decreased immune responses are expected in the elderly and in subjects under dialysis, with central obesity, arterial hypertension, smoking, transplanted patients and patients under immunosuppressive drugs, especially anti-CD20 therapies [6–11]. It is known that vaccination response is reduced in CVID patients [12]. For example, influenza vaccination of individuals with a hypogammaglobulinemia only resulted in significant increase of IgG antibody-titers in 29% [13]. Other studies found an even lower fraction of immune responders after influenza vaccine [14]. Regarding mRNA based vaccines against SARS-CoV-2, several studies show a robust antibody response in the majority of individuals with an antibody deficiency [15–22]. All these findings may lead to the question which patients with humoral immunodeficiencies can be expected to have a good or an insufficient humoral vaccine response. The aim of this study was to further characterize the immune response to mRNA vaccination in relation to the severity of the immunoglobulin deficiency and immunosuppressive medications in an exploratory manner.

## Methods

We studied the humoral immune response against the S1/S2 spike protein of SARS-CoV-2 in patients with humoral immunodeficiency disease. Subjects with a primary or secondary hypogammaglobulinemia (CVID, IgG deficiency, IgG subclass deficiency, drugs, lymphoproliferative disease) and treated by intravenous (IVIG) or subcutaneous immunoglobulin (SCIG) replacement therapy in our outpatient clinic were included if they had received two mRNA Covid-19 vaccine doses (BNT162b2 (Comirnaty®, Pfizer-BionTech) or mRNA-1273 (Spikevax®, Moderna)) 4–6 weeks apart between January and June 2021. CVID was defined with the following characteristics: significant reduced total IgG, reduced IgA or IgM values, poor vaccine response, recurrent infections and absent T-cell impairment [23]. As the production lead time of immunoglobulins is long, it can be assumed, that these products contained no significant amounts of anti-S at the time of the study.

### Data collection and blood sampling

All data on disease, treatment, and vaccinations were collected by the attending physicians based on the medical records. All individual vaccination dates and the vaccines administered were obtained from the original vaccination records in each study participant. Healthy volunteers were recruited among the private and professional surroundings of the investigators without performing a matching procedure. Single serum samples for analysis of vaccine antibodies were collected 2 weeks to 4 months after the second Covid-19 vaccination in all study participants and healthy controls.

### IgG antibody assays

Blood samples were run on an Abbott ARCHITECT i2000 instrument using the Abbott SARS-CoV-2 nucleoprotein assay and the DiaSorin LIAISON® SARS-CoV-2 S1/S2 IgG assay following the manufacturer’s instructions. The Abbott assay is a chemiluminescent microparticle immunoassay for qualitative detection of IgG in human serum or plasma against the SARS-CoV-2 nucleoprotein. The Liaison SARS-CoV-2 S1/S2 is a chemiluminescence assay, that uses paramagnetic microparticles coated with S1 and S2 fragments of the viral surface spike protein (positive cut-off of >12 AU/ml). According to the manufacturer’s information, the Liaison SARS-CoV-2 S1/S2 IgG assay of >15 AU/ml reached a plaque-reduction neutralization test (PRNT) titer of 1:40 in 17/18 patients and a PRNT titer of >1:160 if SARS-CoV-2 S1/S2 IgG of 80 AU/ml were analysed [24]. Presence of antibodies against the nucleocapsid protein (anti-N) were evaluated to capture convalescent persons. The primary endpoint was the proportion of patients, who developed a humoral immune response to SARS-CoV-2 spike protein, compared to healthy controls.

### Ethic approval

This study was approved by the local ethics committee (Kantonale Ethikkommission Bern, ID 2021–01355). All subjects included signed informed consent.

### Statistical analysis

Statistical analysis were performed using Graphpad Prism 9 (GraphPad Software, Inc, La Jolla, Calif). Patient characteristics are summarized with descriptive statistics. Values are median and interquartile ranges (IQR) for continuous variables, categorical variables reported as n (%). The 95% confidence intervals (95%CI) from the proportions of patients with positive anti-S values in a specific group were calculated as Wilson/Brown interval. Anti-S titers were compared by Kruskal-Wallis or Mann-Whitney test, the median 95%CI was calculated for all groups.

## Results

### Patient characteristics

47 patients with a humoral immunodeficiency treated by immunoglobulin replacement therapy and vaccinated twice with one of the Covid-19 mRNA vaccines were identified in a single center. Eight subjects were excluded (4 refused to participate, two had missing anti-S values and two subjects had an interval from the second vaccination to the blood sampling of over 4 months). Of the 39 patients included, 28 were female (71.8%) compared to 15/19 healthy controls (79.0%). The median age was 61 years (IQR 51.0; 73.0) in the study group and 53 years (IQR 43.0; 63.0) in the control group (Table 1). Characteristics of healthy controls are summarized in S1 Table (supporting information). 26/39 subjects (66.7%) fulfilled the criteria of CVID, 2/39 had an IgG deficiency (5.1%), 9/39 an IgG subclass deficiency (mostly combined IgG1/IgG3) (23.1%), one person had a lymphoproliferative disorder and one subject developed a secondary humoral immunodeficiency after ocrelizumab treatment. 36 patients (92.3%) were under IVIG, 3 (7.7%) under SCIG. The median monthly immunoglobulin dose was 25.0 g (IQR 20.0; 30.0). Median total baseline IgG value before initiating immunoglobulin replacement therapy was 5.1 g/l (IQR 3.6; 6.3), median actual IgG trough levels under treatment were 8.9 g/l (IQR 8.1; 10.2). All included patients were twice vaccinated in 4 to 6 weeks intervals, 29/39 (65.6%) with BNT162b2 (Pfizer-Biontech) and 10/39 (31.7%) with mRNA-1273 (Moderna). Anti-N values were negative in all patients, even in two persons with past Covid-19 infection (patient 4 and 27, Table 2). 9/39 patients (23.1%) were under immunosuppressive treatment or had rituximab/ocrelizumab treatment in their past (Table 2).

**Table 1:** Patient characteristics.

	Study participants	Healthy controls
	N = 39	N = 19
**Demographics**		
Age	61.0 (52.5; 73.5)	53.0 (43.0; 63.0)
Gender (female)	29 (70.7%)	15 (79.0%)
Median time from 2. vaccine to sampling (days)	45.0 (33.0; 65.0)	70.5 (46.8; 85.8)
**Clinical**		
CVID (n)	26 (66.7%)	n/a
IgG deficiency (n)	2 (5.1%)	n/a
Secondary immunodeficiency (drugs, neoplastic disease) (n)	2 (5.1%)	n/a
IgG subclass deficiency (n)	9 (23.1%)	n/a
Immunosuppressive drugs (n)	9 (23.1%)	n/a
IVIG (n)	36 (92.3%)	n/a
SCIG (n)	3 (8.3%)	n/a
Monthly dose (g)	25.0 (20.0; 30.0)	n/a
**Laboratory**		
IgA (g/l)	0.58 (0.3; 1.2)	n/a
Baseline IgG before IVIG (g/l)	5.1 (3.6; 6.3)	n/a
IgG nadir value under IVIG/SCIG (g/l)	8.9 (8.1; 10.2)	n/a
SARS-CoV-2-IgG spike protein (AU/ml)	78.9 (12.0; 276.0)	250.0 (209.0; 358.0)

Values are median and interquartile ranges (IQR) for continuous variables. Categorical variables reported as n (%). Laboratory reference values: IgG 7.0–16.0 g/l, IgA 0.7–4.0 g/l, SARS-CoV-2-IgG spike protein cutoff 12 AU/ml.

Common variable immunodeficiency disease (CVID), intravenous immunoglobulin substitution (IVIG), subcutaneous immunoglobulin substitution (SCIG).

**Table 2:** Individual data of included patients.

	Age	Immuno-suppressive drugs	Immuno-deficiency disease	IgA (g/l)	Baseline IgG (g/l)	IgG nadir value under IVIG/SCIG (g/l)	Monthly dose IVIG/SCIG	Vaccine	Time from 2. vaccine to blood analysis (d)	SARS-CoV-2-IgG spike protein (AU/ml)
1	73	No	CVID	<0.05	0.07	8.3	30	BNT162b2	71	<12
2	51	No	CVID	0.07	0.7	7.2	50	mRNA-1273	22	<12
3	55	No	CVID	<0.05	0.8	13.4	28	BNT162b2	39	<12
4	56	No	CVID	1.67	1.9	6.7	25	BNT162b2	40	125
5	57	No	CVID	0.07	1.9	8.2	40	BNT162b2	76	<12
6	56	No	CVID	0.06	3.1	8.2	30	BNT162b2	66	>400
7	72	Ibrutinib / rituximab	secondary	0.7	3.2	10.6	25	mRNA-1273	22	<12
8	86	No	CVID	0.32	3.4	4.9	10	BNT162b2	59	20.4
9	61	No	CVID	<0.05	3.5	10.2	25	BNT162b2	49	14.4
10	49	No	CVID	0.47	3.6	10.6	24	BNT162b2	74	99.9
11	26	No	CVID	0.54	3.8	7.2	70	BNT162b2	22	>400
12	78	Rituximab	CVID	0.49	4.2	8.0	25	BNT162b2	88	<12
13	54	Certolizumab	IgG deficiency	0.83	4.2	6.8	25	BNT162b2	28	37
14	69	No	CVID	11.15	4.3	8.7	25	mRNA-1273	40	372
15	84	No	CVID	0.57	4.3	9.1	30	BNT162b2	112	<12
16	62	Etanercept	IgG deficiency	0.8	4.4	11.4	40	BNT162b2	24	<12
17	74	No	CVID	0.3	4.5	9.3	20	mRNA-1273	63	276
18	86	Rituximab	CVID	<0.05	4.7	8.4	25	BNT162b2	92	<12
19	64	No	CVID	0.53	4.8	8.9	25	BNT162b2	48	59.9
20	59	No	CVID	1.31	5.1	9.6	15	mRNA-1273	22	>400
21	72	No	CVID	1.25	5.3	8.5	50	BNT162b2	34	141
22	91	No	IgG 1 and 3 deficiency	1.23	5.6	7.3	10	BNT162b2	57	109
23	72	Ibrutinib	CVID	0.29	5.7	12	60	mRNA-1273	37	<12
24	31	No	CVID	0.42	5.9	12.1	35	BNT162b2	47	>400
25	31	No	IgG 1 and 3 deficiency	0.92	5.9	9.3	40	BNT162b2	45	>400
26	31	No	CVID	0.58	6	10.0	30	BNT162b2	14	>400
27	72	No	IgG 1 deficiency	1.7	6.1	9.2	25	BNT162b2	62	>400
28	49	No	CVID	0.4	6.2	9.9	40	BNT162b2	22	78.9
29	62	Ocrelizumab	secondary	1.16	6.3	7.8	15	mRNA-1273	33	<12
30	57	Methotrexat / prednisolone	CVID	1.26	6.3	8.3	25	BNT162b2	37	<12
31	23	No	IgG 2 deficiency	0.75	6.5	7.6	15	mRNA-1273	37	136
32	57	No	IgG 1 and 3 deficiency	2.62	6.6	9.6	20	BNT162b2	65	161
33	45	No	CVID	0.33	6.6	8.8	25	BNT162b2	39	248
34	79	No	IgG 1 deficiency	1.18	6.8	9.6	25	mRNA-1273	56	20
35	87	No	IgG 2 and 3 deficiency	2.7	7.5	8.1	10	mRNA-1273	50	>400
36	73	Mycophenolat-mofetile	CVID	0.3	8.3	15.6	30	BNT162b2	43	12.8
37	41	No	IgG 2 deficiency	0.82	8.6	11.2	20	BNT162b2	99	142
38	75	No	IgG 1 and 3 deficiency	2.49	9.1	11.5	20	BNT162b2	71	129
39	58	No	CVID	0.58	5.7	8.7	20	BNT162b2	18	<12

Laboratory reference values: IgG 7.0–16.0 g/l, IgA 0.7–4.0 g/l, SARS-CoV-2-IgG spike protein cutoff 12 AU/ml.

Common variable immunodeficiency disease (CVID), intravenous immunoglobulin substitution (IVIG), subcutaneous immunoglobulin substitution (SCIG).

### SARS-CoV-2 S1/S2 IgG values after two mRNA vaccine doses

**Fig 1:**
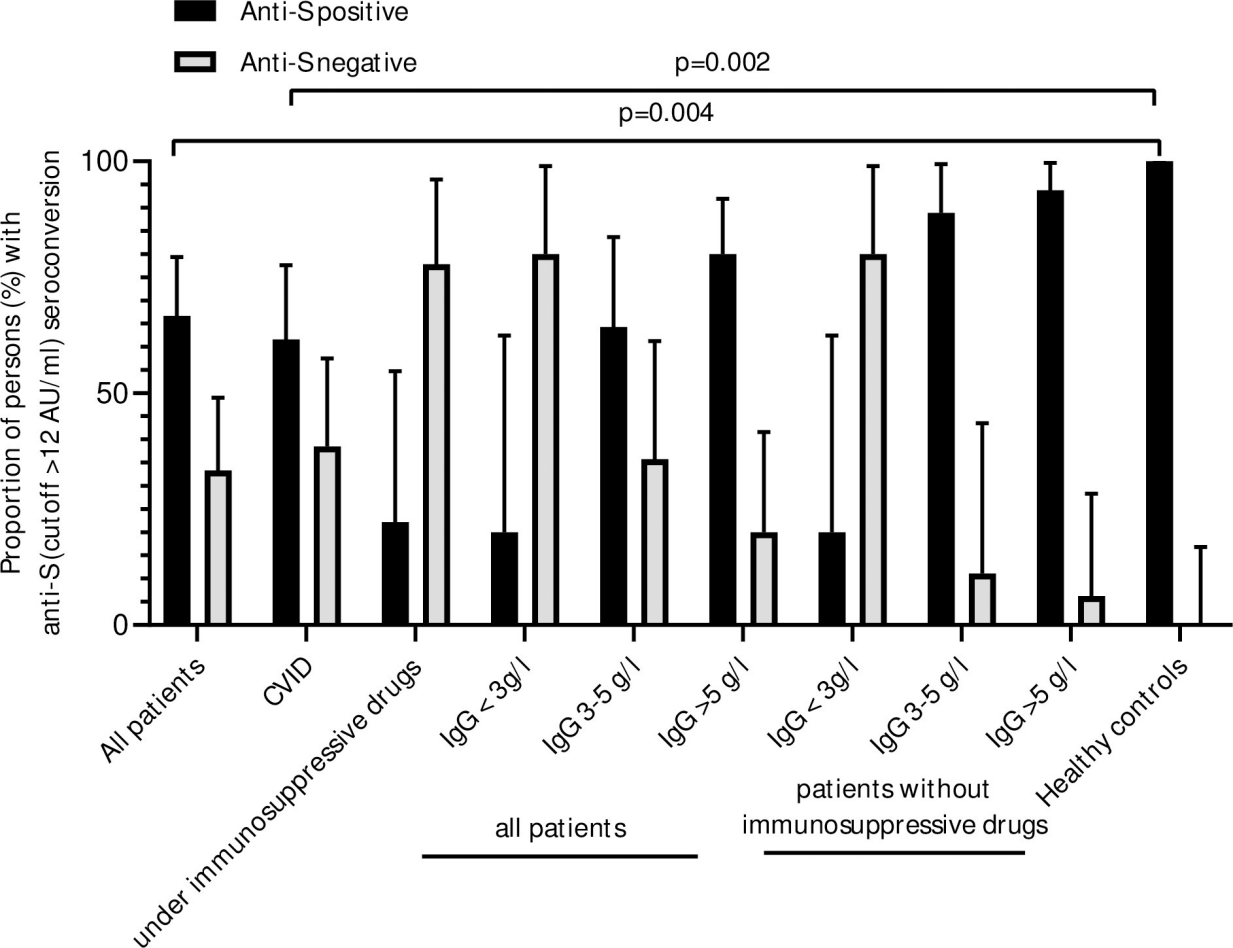
Proportion of patients with anti-S after two Covid-19 mRNA doses. Proportion of patients with humoral immune response to SARS-CoV-2 spike protein (anti-S) are shown in total and for various subgroups (Anti-SARS-CoV-2 spike protein antibodies cutoff >12 AU/ml). Baseline IgG values indicate the severity of the humoral immunodeficiency disease at start of immunoglobulin replacement therapy. P values are calculated by Chi-square test. Anti-SARS-CoV-2 spike protein antibodies (anti-S).

**Fig 2:**
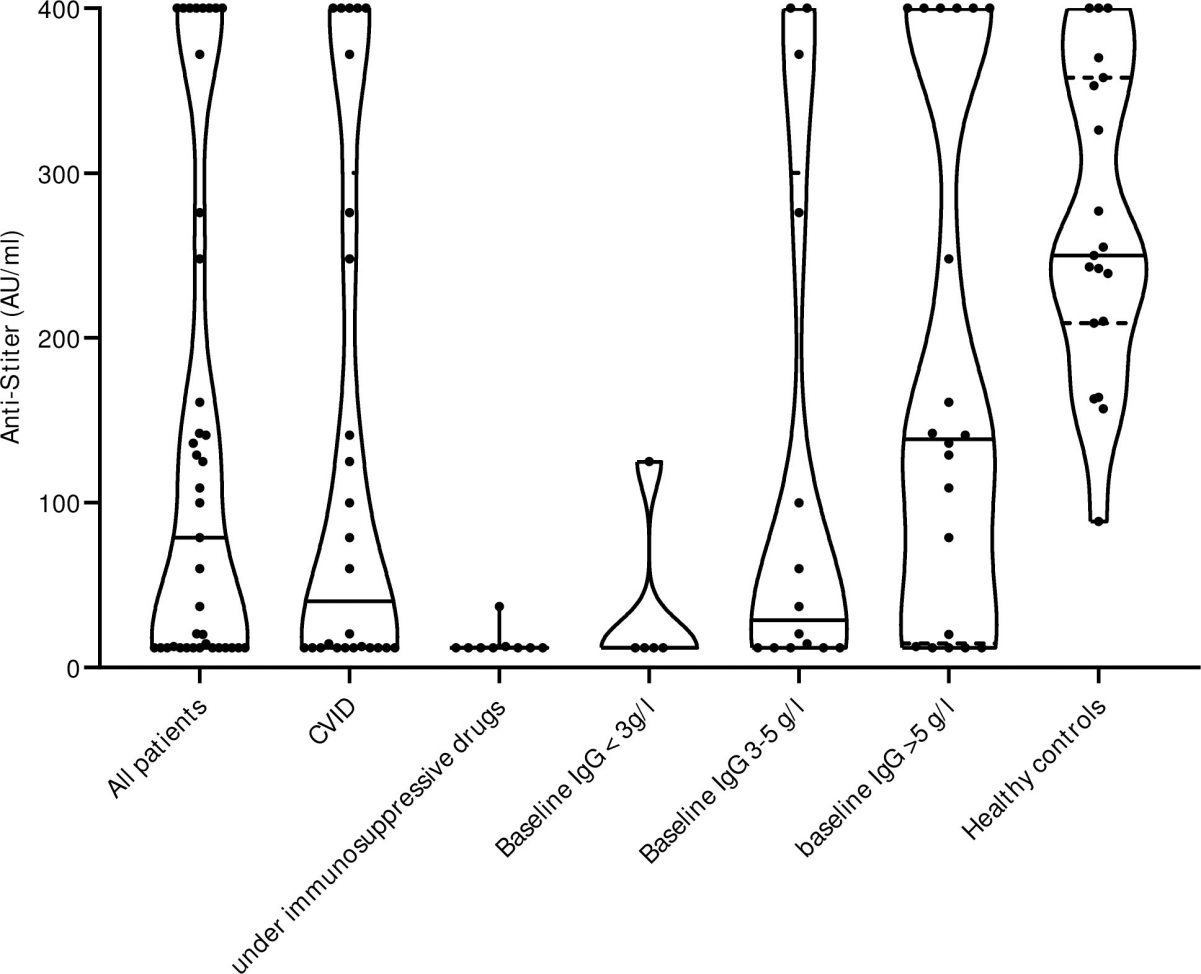
Anti-S quantity subgroup analysis. All included patients were divided into subgroups according to baseline total IgG levels at start of immunoglobulin replacement therapy. Kruskal-Wallis test was calculated for the comparison of the IgG subgroups and healthy control: p = 0.002. Anti-SARS-CoV-2 spike protein antibodies (anti-S).

After two vaccine doses, we found detectable anti-S levels in 26/39 subjects (66.7% [95%CI 51.0–79.4%]) with a humoral immunodeficiency disease, and in all healthy controls (19/19, 100% [95%CI 83.2–100%]). In CVID, the proportion of patients with presence of anti-S was 16/26 (61.5% [95%CI 42.5–77.6%]), and thus lower than in non-CVID patients (3/10, 77.0% [95%CI 49.7–91.8]) and in the healthy control group (19/19, 100.0% [95%CI 83.2–100.0]), p = 0.002. All individuals with a humoral non-response to the vaccines (n = 13) were either under immunosuppressive drugs (including anti-CD20 therapy in the past) (7/13, 53.8%) and/or had a diagnosis of CVID (10/13, 76.9%). Two patients treated by immunosuppressive treatment showed a low anti-S response (Fig 1). Median anti-S concentration in individuals with a humoral immunodeficiency disease was 78.9 AU/ml (95%CI 12.0–142.0), and in the controls 250.0 AU/ml (95%CI 209.0–358.0) (p = 0.002). Patients with CVID had a median anti-S concentration of 40.15 AU/ml (95%CI 12.0–248.0). To assess the correlation of the vaccination response with the severity of the immunodeficiency disease, participating patients were sub-grouped according to baseline IgG levels before the start of immunoglobulin replacement therapy. In subjects without immunosuppressive drugs, only 1/5 patients (20% [95%CI 1.0–62.4%) with a baseline IgG of <3 g/l showed detectable anti-S antibodies, in subjects with baseline IgG between 3–5 g/l 8/9 (88.9% [95%CI 56.5–99.4%) and in the subgroup with IgG values >5 g/l 15/16 (93.8% [95%CI 71.7–99.7%) (Fig 2) showed detectable anti-S. Median anti-S values were significantly lower in patients with severe humoral immunodeficiency disease without immunosuppressive drugs: Baseline IgG <3 g/l: 12.0 AU/ml (95%CI 12.0–125.0), baseline IgG 3–5 g/l: 99.9 AU/ml (95%CI 14.4–400.0), baseline IgG >5 g/l: 151.5 AU/ml (95%CI 109.0–400.0), p = 0.007 (Fig 2). Results were similar when all patients were included in the subgroup analyses (p = 0.002) (Fig 2). Overall, in subjects with baseline IgG >5 g/l there was only a trend to lower anti-S values compared to healthy controls (250.0 AU/ml (95%CI 209.0–358.0)).

### SARS-CoV-2 S1/S2 IgG values after three mRNA vaccine doses

**Fig 3:**
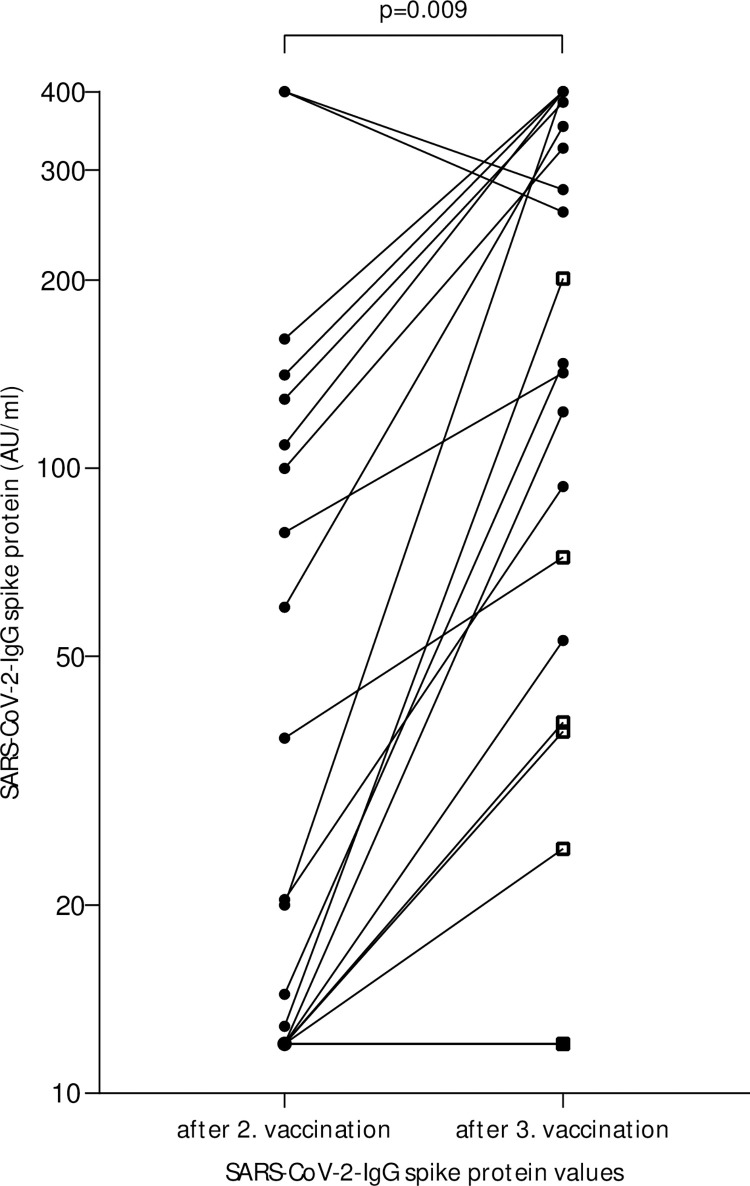
Anti-S quantity after 3. vaccination. Data of SARS-CoV-2-IgG spike protein values of 22 patients with a third mRNA vaccination, including 7 persons under immunosuppressive drugs. In the two subjects with decreasing SARS-CoV-2-IgG spike protein levels despite third vaccination, laboratory analysis was not performed until 4 months after the third vaccination. Subjects under immunosuppressive treatment are marked with square. The p value was calculated by Mann-Whitney test.

In a post-hoc analysis, we obtained anti-S levels from 22 patients after the third vaccination (Fig 3, S2 Table). A third mRNA vaccination was administered in 8/22 subjects with absent anti-S increase after the second vaccination, 7/22 of the patients with a third vaccination were on immunosuppressive drugs. The median anti-S levels significantly increased from 20.2 AU/ml (95%CI 12.0–109.0) to 144.5 (95%CI 39.2–352.0) after the third vaccination (p = 0.009). All but five cases showed an increase in anti-S. Two subjects with preexisting high IgG values showed a slight decrease, but maintained high anti-S antibody levels over time. Reasons for the lack of increase in the other three cases were treatment with immunosuppressive drugs (case 12 and 29) and a CVID disease state (case 39).

## Discussion

26/39 of our patients with humoral immunodeficiency showed a detectable immune response against SARS-CoV-2 after mRNA vaccination. After the second Covid-19 vaccine, the majority of individuals with a mild to moderate IgG deficiency had a specific anti-S concentration that was slightly below the level of healthy controls. Even in most patients with CVID an immune response was detected after a second dose of the Covid-19 mRNA vaccine. Our findings are in line with recent studies, demonstrating an increase of anti-S levels in most individuals with humoral immunodeficiency without immunosuppressive drugs after two Covid-19 vaccinations [15–22]. In a recent study, an immune response to SARS-CoV-2 was detected only in one third of patients with CVID [25]. These data are similar to previous studies on CVID and influenza vaccination [13]. These inconsistent findings may be explained by the severity of the humoral immunodeficiency. Even though immunization with mRNA vaccines is very effective against SARS-CoV-2, it is important to consider confounding factors that may block or attenuate an immune response. In our study, most patients with absent anti-S increase either had a severe form of CVID with a very low IgG baseline titer and/or were currently co-treated by immunosuppressants. This constellation was also observed in a small case series of individuals with CVID by Kinoshita, although these patients developed a significant T-cell response [15]. Nevertheless, patients with humoral immunodeficiency could experience potentially severe breakthrough Covid-19 infections despite vaccination [26]. Importantly, in the majority of those cases with very low or undetectable anti-S levels, an increase was achieved with a third vaccination, in line with the finding of Barmettler et al. [27]. Many country guidelines now advise to administer a third dose in the primary series to patients with CVID, including Swiss guidelines [28].

Therefore, anti-S antibody determination might be useful to detect non-responders after completion of a two Covid-19 vaccination schedule in patients at risk, to enable a timely third vaccination. In patients under immunosuppressive drugs or humoral immunodeficiency, a full vaccination schedule should include three Covid-19 mRNA vaccinations followed by a booster vaccine [29]. If a deficient immune response persists, T-cell assays should be performed and a fourth vaccination should be considered [29].

Our study has limitations: Although both mRNA vaccines seem to be effective in a majority of patients with humoral immunodeficiencies, we have no long-time data since the observational period was very short. Blood samples were taken over a wide time interval (4 weeks to 4 months) in both study groups. Since antibody titers decrease over time, it is therefore likely, that the measured antibody levels underestimate the peak levels in these patients. Furthermore, it has to be assumed that patients with a humoral immunodeficiency have a faster decline of the IgG-anti-S titer than healthy controls. In addition, it is unclear whether the anti-S antibodies we measured are neutralizing in nature. We cannot make any statement about the T-cell response, which certainly plays a major role. Specific T-cell responses seem to be defective in about one third of CVID patients [25]. Lastly, our study groups were small, with both gender and age not completely balanced; this could be a confounding factor. Therefore, prospective cohorts with larger patient numbers and a longer observation period including humoral and cellular vaccine responses are warranted for a better understanding of the long-term effect and benefit of recurrent vaccination in humoral immunodeficiency.

## Conclusion

Although individuals with humoral immunodeficiencies have impaired responses to vaccines, BNT162b2 and mRNA-1273 mRNA vaccines produce humoral immune responses in the majority of these patients. Most individuals with mild to moderate humoral immunodeficiency disease without immunosuppressive drugs did not have significantly reduced anti-S values compared to healthy individuals. Treatment with immunosuppressive drugs and a diagnosis of CVID are risk factors for reduced or even absent humoral response. In these individuals, a full vaccination schedule should include three Covid-19 mRNA vaccinations as primary series followed by a booster vaccine.

## Supporting information

S1 Table SARS-CoV-2-IgG spike protein values of healthy controls. (DOCX)

S2 Table SARS-CoV-2-IgG spike protein (AU/ml) after 3. vaccination. (DOCX)
