# Supplementary material for: Humoral response to mRNA vaccines against SARS-CoV-2 in patients with humoral immunodeficiency disease
Source: PLoS One. 2022 Jun 9;17(6):e0268780. doi: 10.1371/journal.pone.0268780 (PMC9182562; doi:10.1371/journal.pone.0268780)
Supplement: S1 Table — (DOCX) [file pone.0268780.s001.docx]

**S1 table SARS-CoV-2-IgG spike protein values of healthy controls**

|  | Gender | age | Vaccine | SARS-CoV-2-IgG spike protein (AU/ml) | Time from 2. vaccine to blood sampling (d) |
| --- | --- | --- | --- | --- | --- |
| 1 | female | 29 | mRNA-1273 | 250 | 93 |
| 2 | female | 74 | mRNA-1273 | >400 | n/a |
| 3 | female | 30 | mRNA-1273 | 255 | 91 |
| 4 | female | 43 | mRNA-1273 | >400 | 37 |
| 5 | female | 45 | BNT162b2 | 370 | 29 |
| 6 | female | 58 | BNT162b2 | 163 | 99 |
| 7 | female | 41 | BNT162b2 | 243 | 50 |
| 8 | female | 63 | mRNA-1273 | 157 | 76 |
| 9 | female | 53 | mRNA-1273 | >400 | 55 |
| 10 | male | 59 | mRNA-1273 | 277 | 75 |
| 11 | female | 47 | BNT162b2 | 209 | 67 |
| 12 | female | 48 | mRNA-1273 | 239 | 54 |
| 13 | female | 51 | BNT162b2 | 242 | 27 |
| 14 | male | 63 | mRNA-1273 | 88.6 | 74 |
| 15 | male | 61 | mRNA-1273 | 326 | 52 |
| 16 | female | 54 | mRNA-1273 | 358 | 75 |
| 17 | female | 63 | mRNA-1273 | 164 | 84 |
| 18 | female | 33 | mRNA-1273 | 353 | 30 |
| 19 | male | 76 | mRNA-1273 | 210 | 97 |
