# Supplementary material for: Humoral response to mRNA vaccines against SARS-CoV-2 in patients with humoral immunodeficiency disease
Source: PLoS One. 2022 Jun 9;17(6):e0268780. doi: 10.1371/journal.pone.0268780 (PMC9182562; doi:10.1371/journal.pone.0268780)
Supplement: S2 Table — (DOCX) [file pone.0268780.s002.docx]

**S2 Table**

SARS-CoV-2-IgG spike protein (AU/ml) after 3. vaccination

| Patient | SARS-CoV-2-IgG spike protein (AU/ml) after 3. vaccination |
| --- | --- |
| 2 | 53 |
| 7 | 37.9 |
| 8 | 93.5 |
| 9 | 147 |
| 10 | 325 |
| 12 | <12 |
| 13 | 72 |
| 15 | 123 |
| 16 | 39.2 |
| 19 | 352 |
| 21 | >400 |
| 22 | >400 |
| 23 | 24.6 |
| 24 | 279 |
| 26 | 257 |
| 28 | 142 |
| 29 | <12 |
| 32 | >400 |
| 34 | >400 |
| 36 | 201 |
| 38 | 385 |
| 39 | <12 |
